# Prioritization of public health financing, organization, and workforce transformation: a Delphi study in Canada

**DOI:** 10.1186/s12889-023-15373-9

**Published:** 2023-03-22

**Authors:** F. Antoine Dedewanou, Sara Allin, Ak’ingabe Guyon, Jasmine Pawa, Mehdi Ammi

**Affiliations:** 1 School of Public Policy and Administration, Carleton University, Richcraft Hall, 1125 Colonel By Dr, Ottawa, ON K1S 5B6 Canada; 2 Institute of Health Policy, Management and Evaluation, University of Toronto, Toronto, Canada; 3 Department of Epidemiology, Biostatistics and Occupational Health, McGill University, Montreal, Canada; 4 Departement de médecine sociale et préventive, École de santé publique de l’Université de Montréal, Montréal, Canada; 5 Division of Clinical Public Health, Dalla Lana School of Public Health, University of Toronto, Toronto, Canada; 6 Division of Clinical Sciences, NOSM University, Sudbury, Canada; 7 Centre for the Business and Economics of Health, University of Queensland, Queensland, Australia

**Keywords:** Public health systems, Priority setting, Delphi, Thematic analysis, Canada, COVID-19

## Abstract

**Background:**

The increased scrutiny on public health brought upon by the ongoing COVID-19 pandemic provides a strong impetus for a renewal of public health systems. This paper seeks to understand priorities of public health decision-makers for reforms to public health financing, organization, interventions, and workforce.

**Methods:**

We used an online 3-round real-time Delphi method of reaching consensus on priorities for public health systems reform. Participants were recruited among individuals holding senior roles in Canadian public health institutions, ministries of health and regional health authorities. In Round 1, participants were asked to rate 9 propositions related to public health financing, organization, workforce, and interventions. Participants were also asked to contribute up to three further ideas in relation to these topics in open-ended format. In Rounds 2 and 3, participants re-appraised their ratings in the view of the group’s ratings in the previous round.

**Results:**

Eighty-six public health senior decision-makers from various public health organizations across Canada were invited to participate. Of these, 25/86 completed Round 1 (29% response rate), 19/25 completed Round 2 (76% retention rate) and 18/19 completed Round 3 (95% retention rate). Consensus (defined as more than 70% of importance rating) was achieved for 6 out of 9 propositions at the end of the third round. In only one case, the consensus was that the proposition was not important. Proposition rated consensually important relate to targeted public health budget, time frame for spending this budget, and the specialization of public health structures. Both interventions related and not related to the COVID-19 pandemic were judged important. Open-ended comments further highlighted priorities for renewal in public health governance and public health information management systems.

**Conclusion:**

Consensus emerged rapidly among Canadian public health decision-makers on prioritizing public health budget and time frame for spending. Ensuring that public health services beyond COVID-19 and communicable disease are maintained and enhanced is also of central importance. Future research shall explore potential trade-offs between these priorities.

**Supplementary Information:**

The online version contains supplementary material available at 10.1186/s12889-023-15373-9.

## Background

The COVID-19 pandemic has brought significant attention on public health (PH) systems globally. PH systems can be broadly defined as the complex networks of governmental organizations, departments, agencies and associations which are planning, managing and delivering PH services [1, 2]. The COVID-19 crisis has accelerated already engaged critical reflections on PH systems for the 20th century on their infrastructure, scope, processes, outcomes and performance, with Canada being one country particularly active in these reflections since the SARS crisis [3]. Several provincial, territorial, and federal government actors are currently undertaking consultations and reflections to prepare the future of PH systems post-COVID-19 [2, 4–6]. These consultations are yet to produce their full effects in terms of reforms to the Canadian PH systems, as they compete with emerging areas of focus in public health (e.g., public health and climate action) or in healthcare (e.g., funding and surgery backlogs) [7, 8].

Public health systems are understandably complex [9]. In Canada, differing PH responsibilities lie at the federal, provincial/territorial, regional and municipal levels [10]. PH functions include population health assessment, health surveillance, disease and injury prevention, health promotion and health protection [11]. Daily PH interventions at a local or regional level include health needs assessments, direct actions targeted towards populations, indirect actions targeted towards third parties involved in public health response such as support, collaboration and advocacy, as well as planning and evaluation [12]. With pandemic inquiries currently examining PH systems across Canada, multiple priorities will be proposed to allow for concrete actions for renewal of PH systems.

Health systems are particularly known to be slow and incremental to reform, before windows of opportunity for large-scale, substantial reforms open [13]. The COVID-19 pandemic provides this kind of window of opportunity for major reform to PH systems, with both opportunities and risks. Collecting data from PH senior decision-makers in this setting, and using a method designed to build consensus where possible and identifying consistent needs across jurisdictions, is thus a particularly timely endeavor.

Given the underdeveloped field of PH systems and services research [14], and despite several policy reports on reforming PH systems, PH decision makers’ priorities on PH systems are seldom systematically investigated and documented. This study provides a first step in this direction and will help highlight priorities for both PH policies and intervention research. Our objectives are to understand PH decision makers’ priorities for PH systems reform across Canada, and ultimately generate evidence on PH decision-makers expectations for future PH systems’ reorganizations. Precisely, our study aims to identify if a consensus can emerge on priorities for public health financing, organization, interventions, and workforce across senior PH decision makers located in several jurisdictions and organizations across Canada.

## Methods

### Research design

We developed and administered a Delphi survey to collect data from senior PH decision makers. The Delphi approach is a method for organizing and sharing opinions among a panel of experts with the aim to identify areas of consensus [15–17]. Experts are asked to make judgments, usually via a rating of importance, on a list of ideas which are called propositions in the Delphi terminology. The Delphi approach is iterative, and the rating of importance is repeated by one expert on several occasions called rounds. Because the purpose is potential consensus building, the experts are presented with the ratings of the group between each round [18–20] and asked to reconsider their ratings in the next rounds based on this new information. We used a type of real-time Delphi in an online survey characterized by participants being able to participate and edit their responses anytime during a specific timeframe [21].

The first step of a Delphi is thus to develop a list of propositions. We started by conducting a literature review to identify key components of public health infrastructures [22–24] and we consulted with a working group of PH experts, including PH academic, PH practitioners and clinicians, and PH managers, to develop an initial list of propositions summarizing important characteristics of PH infrastructure in Canada. We then refined our list of propositions in three consultations with this working group of PH experts and ran a pilot test of the first round of the Delphi survey with 3 participants. We then aggregated our proposition lists from 16 to 9 propositions due to overlap in themes. The 9 propositions cover the domains of PH financing, organization, workforce, and interventions, all well-identified as key dimensions in the literature of PH systems [22–24]. These specific propositions were also consistent with recent PH systems reorganizations in various Canadian jurisdictions [1] and with the key messages that emerged in the recent Canadian Chief Public Health Officer’s report [2]. Additionally, we added an option for participants to provide open-ended comments on up to three priorities, to be analyzed separately. We included this option to provide nuance to the findings of the Delphi.

The second step was to determine the number of Delphi rounds, a debated issue in the literature [20, 25]. On one hand, some recommend that the appropriate number of rounds should be determined once the panelists show a level of stability in their individual responses [19, 20, 25]. On the other hand, some scholars argue that practical constraints such as time, cost, and expert availability require a set number of Delphi rounds. Since three rounds are generally considered sufficient to reach group consensus [18, 20, 26], we elected for a fixed three-round data collection given the expected constraint on PH experts’ availability.

The Delphi process was conducted in an online format, as all real-time Delphi, and owing to the COVID-19 pandemic context. Note that online Delphi performs similarly to in person [18, 21, 27, 28].

### Survey instruments

Participants were prompted to identify their preferences for changes to public health financing and structures in order to determine emerging priority areas and help inform the future changes for public health systems in Canada. They were asked to rate the nine propositions on a 5-point Likert scale (“not at all important”, “slightly important”, “moderately important”, “very important”, “extremely important”). Propositions in the financing domain include provincial or territorial PH budget, sources of PH financing, and time frame for spending. For the domain of PH organization, propositions concern centralization or decentralization of public health system, integration of public health with other health sectors, and the creation of public health structures with specialized public health functions. Further, we assess participants’ opinions regarding the disciplinary skill-mix of PH human resources. Our last domain covers PH interventions related to the COVID-19 pandemic and those which are not related to the COVID-19 pandemic. We provide the full description of our questionnaire in the Supplementary Materials. In Round 2, we show individual and aggregate responses to each participant and invite them to re-rate the nine propositions with the possibility to re-consider their initial responses. They receive the same instructions during the Round 3.

The Delphi survey was developed online using Qualtrics. The survey was initially developed in English and then translated in French by our bilingual team members: one researcher translated the survey to French, and another translated it back to English to ensure equivalence. Only minor adjustments were made to the initial French translation. An important feature of our survey design is that, after round 1, each respondent receives an individualized survey. Indeed, in round 2, each respondent receives the results summarizing both their own response and the overall responses on the importance of each of the 9 propositions at the end of the first round. This is with the view to minimize survey recall bias and inform the respondent of the collective priorities. With this new information, the respondents are invited to re-rate the 9 propositions with the possibility to re-consider their initial responses. The same process applies in Round 3, displaying the individual and group responses of the second round.

### Participants’ recruitment

The literature on the number of participants required in a Delphi exercise suggests many acceptable ranges, from 5 to 50 participants [29–31]. The lower end of these ranges have been used in empirical studies [32–34] and most recently, Vogel et al. [35] argued that 12 respondents is a sufficient minimum. In line with Belton et al. [18] and Rowe and Wright [20], we invited 86 PH decision makers to participate in the Delphi survey. We aimed for 15 to 20 respondents in the last round and our number of first invitations account for potentially high initial non-response and for some attrition between rounds.

Our target population was of individuals in senior position in a Canadian public health organization (e.g., provincial public health organization, local public health unit) or a health organization with a public health mandate (e.g., Ministry of Health, regional health authority). The rationale for focusing on senior decision-makers was for them to be able to directly influence or act on priorities for PH infrastructure and to address gaps in the literature in understanding this perspective. This rationale led us to exclude academics from our target population. Senior roles include medical officers of health, senior manager, or senior policy advisors in these organizations. We aimed for a national coverage and invited respondents from all provinces and territories. We invited respondents working in provincial/territorial and regional authorities to account for the multi-level nature of PH systems. We built a directory from publicly available information, supplemented by input from knowledge users for those harder to locate. We adopted a non-probability purposive sample strategy to ensure invited participants met our inclusion criteria.

Respondents were invited to participate by email sent to their professional address in April 2021. To increase participation, we asked our knowledge users to champion the Delphi in their province/territory by sending a pre-invitation email highlighting our invitation was coming. The data collection occurred from April to June 2021, only those who received the individual invitation could access their personalized survey, and only respondents who replied to the preceding round received invitation for the next one.

### Reaching consensus

There is no agreed position in Delphi literature concerning the way that researchers must define and operationalize consensus among the participants [19, 26, 36–38]. Some recommend using both stability and consensus on a round-by-round basis and continuing until acceptable levels of both are achieved [18, 19]. Consensus criteria can include the same or similar opinion being reported by 70% [35, 36, 39, 40], 75% [29, 41] or 80% of experts [31, 42]. In this paper, we define consensus as a minimum of 70% of participants’ agreement on the importance of the proposition. We selected this lower bound given the heterogenous participants coming from various organizations and geographic levels with goals potentially not aligned. Participants are deemed to agree on a proposition’s importance if they are at least 70% to judge it as very/extremely important. They agree on a proposition being not important if they are at least 70% to rate it as not/slightly/moderately important.

### Data analysis

We perform descriptive statistics to describe respondents’ characteristics and group responses to each proposition in all three Delphi rounds. Analyses are conducted using STATA SE 16. Open-ended comments are synthesized using thematic analysis [43]. Themes were organized through discussion, summary tables, and mapping.

## Results

### Participants description

Of the 86 participants invited to take part the Delphi study, 25 experts completed Round 1 (29% response rate), 19 of 25 completed Round 2 (76% retention rate) and 18 of 19 completed Round 3 (95% retention rate). The dropout rate between each round is thus consistent with the health related Delphi literature [35, 39]. Table 1 presents the demographic characteristics of participants in each round. At the beginning of the survey 20% of participants received the French version of the questionnaire while 80% participated in English. Respondents of all the parts of Canada were represented, roughly proportional to the population and with slightly fewer responses from the territories, as expected. Most participants are non-MOH executive, who responded consistently across rounds, while the share of CMOH and MOH decreased from the first to the last round. Respondents are slightly more represented in Ministries of Health, and relatively evenly spread between Public Health Agencies and Regional Health Authorities.

**Table 1:** Characteristics of the participants

	Round 1	Round 2	Round 3
	n = 25	n = 19	n = 18
**Province/Territory**			
British Columbia (BC)	32%	31.6%	27.8%
Atlantic (NB, NL, NS, PEI)	16%	10.5%	11.1%
Ontario (ON)	16%	21%	22.2%
Prairies (AB, MB, SK)	12%	10.5%	11.1%
Quebec (QC)	16%	21%	22.2%
Territories (NT, NU, YK)	8%	5.3%	5.5%
**Survey language**			
English	80%	73.7%	72.2%
French	20%	26.3%	27.8%
**Current Role**			
CMOH, MOH and equivalents	44%	47.4%	44.5%
Executive non-MOH	56%	52.6%	55.5%
**Organization**			
Ministry of Health	40%	47.4%	44.4%
Public Health Organization	36%	31.6%	27.8%
Local or Regional Health Authority	24%	21%	27.8%

Note: AB: Alberta; MB: Manitoba; NB: New Brunswick; NL: Newfoundland-Labrador; NS: Nova Scotia; NT: Northwest Territories; NU: Nunavut; PEI: Prince Edward Island; SK: Saskatchewan; YK: Yukon

### Delphi main findings

**Fig. 1:**
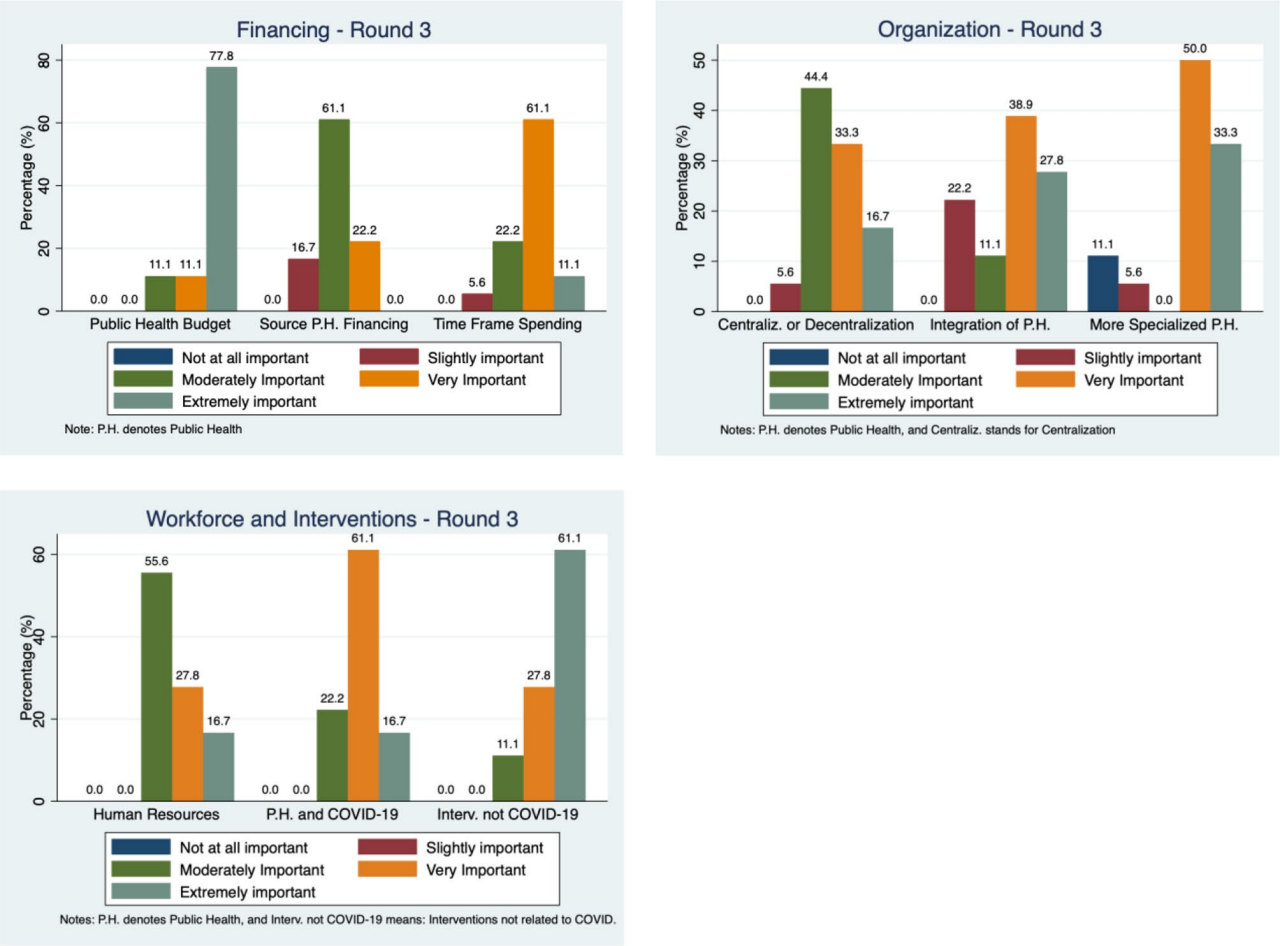
Participants’ responses during the third round of the Delphi survey

Table 2 presents a summary of the importance ratings for all Delphi propositions across the three rounds of data collection. Recall ratings as “very important” or “extremely important” are aggregated in the “important” category, while the other levels of the Likert scale fall in the “not important” aggregated category, and we provide more details of the full distribution of the ratings for Round 3 in Fig. 1. The numbers in Table 2 reports the percentage of respondents who judge the proposition as important or not important, and figures in bold indicate that the consensus has been reached for the proposition.

The number of propositions where consensus was achieved increased for several propositions from Round 1 to Round 3. In Round 1, consensus was achieved for 2 of the 9 propositions. In Round 2, consensus was achieved for 4 of the 9 propositions and this rose to 6 out of 9 in Round 3. By Round 3, consensus was achieved on the importance of: public health budget (89% judge it important in round 3); time frame for spending (72%); public health structures with specialization of public health functions (83%); public health interventions related to the COVID-19 pandemic (78%); and PH interventions not related to the COVID-19 pandemic (e.g., environmental health protection, prevention and control of other infectious diseases) (89%). The proposition pertaining to the source of PH financing is the only one where the consensus led to a rating of non-importance (78% of respondents judge it unimportant).

Turning to Fig. 1, it appears that among propositions where consensus was reached, majority judgment of extreme importance was only attained for PH budgets and PH interventions not related to COVID-19. The other consensual propositions (time frame for spending, PH specialization, COVID-19 PH interventions) were rated as very important.

**Table 2:** Responses to the propositions across the three rounds

	Round 1 (n = 25)		Round 2 (n = 19)		Round 3 (n = 18)	
	Important %	Not important %	Important %	Not important %	Important %	Not important %
1. Public health budget in your province or territory (e.g., increase, decrease or stability)	**88**	12	**94.7**	5.3	**88.9**	11.1
2. Source of public health financing (e.g., mostly federal or mostly provincial or mostly municipal)	44	56	47.3	52.7	22.2	**77.8**
3. Time frame for spending (e.g., restricted to a fiscal year or possible to use beyond a fiscal year)	64	36	**84.2**	15.8	**72.2**	27.8
4. Centralization or decentralization (e.g., less or more public health structures in your province or territory)	68	32	52.6	47.4	50	50
5. Integration of public health with other health sectors (e.g., with primary care)	52	48	57.8	42.2	66.7	33.3
6. Creation of public health structures with more specialized public health functions (e.g., some focus on surveillance, other on promotion)	60	40	68.4	31.6	**83.3**	16.7
7. Disciplinary skill-mix of public health human resources (e.g., concentration of the workforce mostly in some disciplines or workforce trained in more disciplines)	48	52	42.1	57.9	44.4	55.6
8. Public health interventions related to the COVID-19 pandemic (e.g., surveillance, case and contact management, infection prevention and control, risk communication)	64	36	**73.7**	26.3	**77.8**	22.2
9. Public health interventions not related to the COVID-19 pandemic (e.g., environmental health protection, prevention and control of other infectious diseases)	**88**	12	**94.7**	5.3	**88.8**	11.2

Bold % denotes that 70% consensus was achieved

### Synthesizing open-ended comments provided

Free-text suggestions were solicited during Round 1 of the Delphi process and most participants provided comments. Most of the comments touched on governance and decision-making, the importance of financing/resourcing and human resources. Less common comments related to data and information systems. In sum, in addition to the domains covered in the survey (i.e., financing and workforce), two other themes emerged (i.e., governance and information system).

“Ongoing committed long-term funding rather than responsive funding to address each emerging public health crisis. This allows long-term planning and optimization as well as better prevention.”

Regarding PH financing, survey respondents noted the need to increase (and protect) the PH budget over the long-term, to increase funding for and consolidation of public health laboratories, to allow for flexibility within PH budgets in terms of the different PH functions, and to consider equity in the funding formulas to reflect differences in local populations’ health and vulnerabilities. Key priorities noted within the PH workforce domain included the need to expand and “upskill” the PH workforce and investing in training and increased compensation.

“The expert public health workforce has been demoralized during the pandemic response, decimating available expertise. Investment in training, improved compensation, and most importantly basic respect for front line public health expertise is urgently required.”

Many of the open-text comments related to public health governance. Some of the comments touched on aspects of governance and decision making around financing and workforce, such as the need to develop a strategy and specific objectives for PH to use to then clarify and justify the budgets and resources, as well as the need to develop a workforce strategy. Multiple individuals commented on the need for increased clarity of public health objectives and the inherent challenges given the nature of the work. Some reflected on the need to prioritize more non-communicable disease work (over communicable disease) and continue to work towards prioritizing prevention/promotion over treatment, an aspect covered in the survey. Other aspects of governance noted in the comments included maintaining or increasing the independence of PH actors, with some noting the importance of maintaining independence from the acute care system, as well as supporting intersectoral partnerships and including multiple stakeholders (e.g., from social sectors) in decision making. Some indicated a lack of respect for PH expertise, particularly that of local/regional PH leadership.

“Governance of public health and its independence from the administrative functions of government. Accountability of public health with respect to funding, organization of services and personnel.”

A last and smaller group of comments pertained to the importance of data management and information systems. They reflected on epidemiologically driven nature of public health and the need for improved availability of data for PH to continue advancing its work and the science.

## Discussion

### Key findings

Using a Delphi methodology, we investigated PH senior level decision-makers priorities for the future of PH infrastructure in Canada. The development of the Delphi survey in close consultation with PH knowledge users brought us to focus on PH financing, organization, workforce, and interventions. The relevance of this focus was recently corroborated by priority research areas identified by the CIHR Institute of Population & Public Health [44]. Consensus was reached on six out of nine propositions. One of the most important findings is the immediate consensus in the first round of the Delphi, and agreement on extreme importance among PH decision-makers on the importance of PH budget and PH non-COVID-19 interventions. Several have observed cuts in PH budgets [45, 46], even if there are variations across Canada [47]. The need for sufficient financial means to support their work is thus a top priority, and PH decision-makers are also mindful that the COVID-19 pandemic shall not crowd-out all the other necessary PH interventions. With regards to non-COVID public health interventions needed to be prioritized, multiple comments specifically pointed at the importance of upstream and health promotion interventions. Indeed, public health institutions have faced a recurring challenge in simultaneously addressing population health needs and public health emergencies [6, 48]. Nevertheless, PH decision-makers also agree that PH interventions designed to tackle the COVID-19 pandemic are important, perhaps recognizing that the implications of the pandemic will be far reaching.

Among other propositions reaching consensus, the time to spend the PH budget is important but the source of financing is not. While the source of financing could reflect a division of power in the way the money is spent, most jurisdictions receive funding mostly from provincial and federal governments. The main exception is the province of Ontario, where the municipal level provides a nonnegligible share of the funding [49]. Hence, the little variation they experience in the source of financing may explain why PH decision-makers from across the country do not see this as a priority. On the other hand, the time frame to spend the PH budget may be related to the long-term planning and implementation horizon of many PH interventions. Creating PH structures with more specialized PH functions also reached consensus. Most provinces and territories do not have regional PH expertise centers (e.g., BC Centre for Disease Control, Public Health Ontario, Institut National de la Santé Publique du Québec) [49–52], and the consensus may reflect a desire to protect or expand those centers.

Where consensus was not reached, it was for the centralization/decentralization of PH, PH integration and the skill-mix of the PH workforce. Canadian health systems, including PH, have increasingly recentralized from the mid-2000s, but there is no clear evidence on the right level of centralization/decentralization [53]. Integration of PH with other healthcare sectors, particularly primary care, is recognized as a way to improve population health [54]. However, only some provinces like Quebec have been moving in this direction [55]. Moreover, while there are clearly established core competencies for PH in Canada [56], little is known on the size and composition of the Canadian PH workforce [49, 51]. The dearth of evidence on these matters may help explain why consensus was not reached, even though some comments in the open text pertained to skill-mix.

Overall, free-text comments provided by participants in the first round of the Delphi process provided useful and complementary insights, particularly on the critical importance of public health governance. PH governance can be understood as “the ways in which public, non-governmental, or private actors work together to support communities in preventing disease and achieving health, wellbeing, and health equity” [57]. Functions of PH governance include resource stewardship [58], which emerged as an important theme in the comments.

### Strengths and limitations

Our study has several strengths. We focused on PH senior decision makers rarely involved in anonymous, consensus-building processes. This approach should limit issues that may occur in group consultations where authority, personality or reputation may affect priority-setting. Obtaining the perspective of individuals likely to be making or influencing policy decisions is an important contribution. We further achieved a good coverage of multiple level of responsibilities, from local to provincial. Furthermore, given its reasonable time-demand, the online Delphi may have been the most realistic strategy to survey PH decision-makers during a pandemic. We used a bilingual survey, ensuring representation of linguistic minority in Canada, and covered the country coast to coast to coast.

Our study also has limitations. We obtained an uneven initial response rate across jurisdictions, which may be explained by differential timing of COVID waves across the country. However, we achieved high retention rate among the respondents. We aimed for a short list of propositions to maximize the survey uptake, hence not all areas of PH infrastructure were addressed. Further, we did not change the propositions between each Delphi round. We aimed to mitigate this by offering the option for open-ended comments, which helped reveal the importance of public health governance as well as data management and information systems. Lastly, the formulation of our propositions did not allow us to know if changes related priorities identified by participants were already being implemented.

Future research could delve into those priorities with more extensive qualitative methods to consolidate and refine concrete policy options. This could be done by expanding the investigation to larger PH community (e.g., academics, PH staff). It would also be useful to know if these priorities resulted in actual system changes. Additionally, a quantitative study could help clarify the potential trade-offs among priorities.

## Conclusion

Public health systems renewal is now at the top of many policy agendas. Ensuring that their redesign is informed by public health communities will not only increase the readiness of the systems for future public health challenges, but they will also increase their acceptability by those in charge of transforming the public health systems. Despite the difficulty of obtaining participation from public health leaders in the middle of the COVID-19 pandemic, we attained the required number of responses for the Delphi methodology and achieved a geographic coverage of Canada. The top priorities of PH senior decision-makers are PH budget and ensuring non-COVID PH interventions are not forgotten because of the pandemic. Future research can help assess if and how PH decision makers are willing to accept trade-offs between these priorities.

## Electronic supplementary material

Below is the link to the electronic supplementary material.

Supplementary Material 1

## Data Availability

The dataset generated and/or analyzed during the current study are not publicly available to protect the privacy of the respondents but are available from the corresponding author on reasonable request.

## List of abbreviations

AB: Alberta
BC: British Columbia
COVID-19: Coronavirus disease
CMOH: Chief Medical Officer of Health (also sometimes referred to as Chief Public Health Officer)
MB: Manitoba
MOH: Medical Officer of Health (also sometimes referred to as Medical Health Officer)
NB: New Brunswick
NL: Newfoundland-Labrador
NS: Nova Scotia
NT: Northwest Territories
NU: Nunavut
PEI: Prince Edward Island
PH: Public health
QC: Quebec
SK: Saskatchewan
ON: Ontario
YK: Yukon

## References

[1] Canadian Public Health Association. Public health in the context of health system renewal in Canada. Background Document. 2019. Disponible sur: https://www.cpha.ca/sites/default/files/uploads/policy/positionstatements/phhsr-backgrounddocument-e.pdf

[2] Public Health Agency of Canada. A vision to transform Canada’s public health system. Chief Public Health Officer’s Report on the State of Public Health in Canada 2021. Ottawa, ON: Public Health Agency of Canada. 2021 déc. p. 129. Disponible sur: https://www.canada.ca/en/public-health/corporate/publications/chief-public-health-officer-reports-state-public-health-canada/state-public-health-canada-2021.html

[3] Public Health Agency of Canada, the National Advisory Committee on SARS and Public Health. Learning from SARS: Renewal of public health in Canada – Report of. 2004. Disponible sur: https://www.canada.ca/en/public-health/services/reports-publications/learning-sars-renewal-public-health-canada.html

[4] Canadian Institutes of Health Research, Institute of Population and Public Health. Building public health systems for the future. Dialogue Background Paper. Apr 2021. Disponible sur: https://cihr-irsc.gc.ca/e/52413.html

[5] Commissaire à la santé et au bien être. Le devoir de faire autrement - Partie 1: Renforcer le rôle stratégique de la santé publique [Internet]. Quebec. ; 2022 janv p. 102. Disponible sur: https://www.csbe.gouv.qc.ca/fileadmin/www/2022/Rapportfinal_Mandat/CSBE-Rapport_final_Partie1_SP.pdf

[6] DeSalvo K, Hughes B, Bassett M, Benjamin G, Fraser M, Galea S et al. Public health COVID-19 impact assessment: lessons learned and compelling needs. NAM Perspectives. 7 avr 2021. Disponible sur: https://nam.edu/public-health-covid-19-impact-assessment-lessons-learned-and-compelling-needs/10.31478/202104c PMC8406505 34532688

[7] Public Health Agency of Canada. Public Health Agency of Canada. Chief Public Health Officer of Canada’s Report on the State of Public Health in Canada 2022: Mobilizing Public Health Action on Climate Change in Canada. [Internet]. Ottawa. ; 2022 déc p. 103. Disponible sur: https://www.canada.ca/content/dam/phac-aspc/documents/corporate/publications/chief-public-health-officer-reports-state-public-health-canada/state-public-health-canada-2022/report-rapport/report.pdf

[8] Major D, CBC News. Premiers accept federal health-care funding proposal | CBC News [Internet]. 2023 [cité 16 févr 2023]. Disponible sur: https://www.cbc.ca/news/politics/premiers-accept-federal-health-proposal-1.6746976

[9] Rechel B, Maresso A, Sagan A, Hernández-Quevedo C, Williams G, Richardson E et al. éditeurs. Organization and financing of public health services in Europe: Country reports. Copenhagen (Denmark): European Observatory on Health Systems and Policies. 2018. (European Observatory Health Policy Series). Disponible sur: http://www.ncbi.nlm.nih.gov/books/NBK507325/29897704

[10] Marchildon G, Allin S, Merkur S. Health systems in transition: Canada. 3rd ed. Toronto, On: University of Toronto Press; 2021. p. 240.

[11] WHO. Self-assessment tool for the evaluation of essential public health operations in the WHO European Region. World Health Organization. 2015 p. 113. Disponible sur: https://www.euro.who.int/__data/assets/pdf_file/0018/281700/Self-assessment-tool-evaluation-essential-public-health-operations.pdf

[12] Litvak E, Dufour R, Leblanc É, Kaiser D, Mercure SA, Nguyen CT (2020). Making sense of what exactly public health does: a typology of public health interventions. Can J Public Health.

[13] Tuohy CH. Remaking policy: scale, pace and political strategy health care reform. Toronto, On: University of Toronto Press; 2018. p. 688.

[14] Scutchfield FD, Lawhorn N, Ingram R, Péerez DJ, Brewer R, Bhandari M (2009). Public Health Systems and Services Research: dataset development, dissemination, and use. Public Health Rep.

[15] Bardecki MJ (1984). Participants’ response to the Delphi method: an attitudinal perspective. Technol Forecast Soc Chang.

[16] Keeney S, Hasson F, McKenna HP (2001). A critical review of the Delphi technique as a research methodology for nursing. Int J Nurs Stud.

[17] Krucien N, Le Vaillant M, Pelletier-Fleury N (2013). Do the organizational reforms of general practice care meet users’ concerns? The contribution of the Delphi method. Health Expect.

[18] Belton I, MacDonald A, Wright G, Hamlin I (2019). Improving the practical application of the Delphi method in group-based judgment: a six-step prescription for a well-founded and defensible process. Technological Forecast Social Change 1 oct.

[19] Heiko A (2012). Consensus measurement in Delphi studies: review and implications for future quality assurance. Technol Forecast Soc Chang.

[20] Rowe G, Wright G. Expert opinions in forecasting: the role of the Delphi technique. Principles of forecasting.Springer; 2001.pp. 125–44.

[21] Aengenheyster S, Cuhls K, Gerhold L, Heiskanen-Schüttler M, Huck J, Muszynska M (2017). Real-time Delphi in practice — a comparative analysis of existing software-based tools. Technological Forecast Social Change 1 mai.

[22] Handler A, Issel M, Turnock B (2001). A conceptual Framework to measure performance of the Public Health System. Am J Public Health août.

[23] Meyer AM, Davis M, Mays GP (2012). Defining organizational capacity for public health services and systems research. J Public Health Manag Pract nov.

[24] Guyon A, Perreault R (2016). Public health systems under attack in Canada: evidence on public health system performance challenges arbitrary reform. Can J Public Health mai.

[25] Erffmeyer RC, Erffmeyer ES, Lane IM (1986). The Delphi technique: an empirical evaluation of the optimal number of rounds. Group & organization studies.

[26] Boulkedid R, Abdoul H, Loustau M, Sibony O, Alberti C (2011). Using and reporting the Delphi Method for Selecting Healthcare Quality Indicators: a systematic review. PLOS ONE juin.

[27] Gary JE, Heiko A (2015). The future of foresight professionals: results from a global Delphi study. Futures.

[28] Gordon T, Pease ART, Delphi (2006). An efficient,“round-less” almost real time Delphi method. Technol Forecast Soc Chang.

[29] Hasson F, Keeney S, McKenna H (2000). Research guidelines for the Delphi survey technique. J Adv Nurs.

[30] Rowe G, Wright G. Expert opinions in forecasting: the role of the Delphi Technique. In: Armstrong JS, éditeur. Principles of forecasting: a handbook for researchers and practitioners. Boston, MA: Springer US; 2001. p. 125–44. (International Series in Operations Research & Management Science).

[31] Toma C, Picioreanu I (2016). The Delphi technique: methodological considerations and the need for reporting guidelines in medical journals. Int J Public Health Res.

[32] Boje DM, Murnighan JK (1982). Group confidence pressures in iterative decisions. Manage Sci.

[33] Brockhoff K. IV. E. The performance of forecasting groups in computer dialogue and face-to-face discussion. Volume 68. Addison Wesley Publishing Company; 1975.

[34] Belton I, Wright G, Sissons A, Bolger F, Crawford MM, Hamlin I et al. Delphi with feedback of rationales: How large can a Delphi group be such that participants are not overloaded, de-motivated, or disengaged? Technological Forecasting and Social Change. 1 sept 2021;170:120897.

[35] Vogel C, Zwolinsky S, Griffiths C, Hobbs M, Henderson E, Wilkins E (2019). A Delphi study to build consensus on the definition and use of big data in obesity research. Int J Obes.

[36] Diamond IR, Grant RC, Feldman BM, Pencharz PB, Ling SC, Moore AM (2014). Defining consensus: a systematic review recommends methodologic criteria for reporting of Delphi studies. J Clin Epidemiol 1 avr.

[37] Hsu CC, Sandford BA. The Delphi Technique: Making Sense of Consensus. Practical Assessment, Research, and Evaluation [Internet]. 2007 [cité 19 sept 2020];12(10). Disponible sur: https://scholarworks.umass.edu/pare/vol12/iss1/10/

[38] Mitchell VW. The delphi technique: an exposition and application. null. 1 janv 1991;3(4):333–58.

[39] Henderson EJ, Rubin GP (2012). Development of a community-based model for respiratory care services. BMC Health Serv Res.

[40] Slade SC, Dionne CE, Underwood M, Buchbinder R. Standardised method for reporting exercise programmes: protocol for a modified Delphi study.BMJ open. 2014;4(12). 10.1136/bmjopen-2014-006682 PMC4281530 25550297

[41] Keeney S, Hasson F, McKenna H (2006). Consulting the oracle: ten lessons from using the Delphi technique in nursing research. J Adv Nurs.

[42] Green B, Jones M, Hughes D, Williams A (1999). Applying the Delphi technique in a study of GPs’ information requirements. Health Soc Care Commun.

[43] Braun V, Clarke V (2006). Using thematic analysis in psychology. Qualitative Res Psychol 1 janv.

[44] Institute of Population and Public Health. Moving forward from the COVID-19 pandemic: 10 opportunities for strengthening Canada’s public health systems. [Internet]. Ottawa: Canadian Institutes of Health Research. ; 2022 mars p. 34. Disponible sur: https://cihr-irsc.gc.ca/e/documents/ipph-10-opp-report-en.pdf

[45] Caldwell HAT, Scruton S, Fierlbeck K, Hajizadeh M, Dave S, Sim SM et al. Fare well to Nova Scotia? Public health investments remain chronically underfunded. Can J Public Health. 2021;112(2):186–190. 10.17269/s41997-021-00478-8 10.17269/s41997-021-00478-8 PMC7903927 33625685

[46] Fiset-Laniel J, Guyon A, Perreault R, Strumpf EC. Public health investments: neglect or wilful omission? Historical trends in Quebec and implications for Canada. Can J Public Health. 2020;111(3): 383–388. 10.17269/s41997-020-00342-1 10.17269/s41997-020-00342-1 PMC7278767 32514719

[47] Ammi M, Arpin E, Allin S (2021). Interpreting forty-three-year trends of expenditures on public health in Canada: long-run trends, temporal periods, and data differences. Health Policy 1 déc.

[48] Khan Y, O’Sullivan T, Brown A, Tracey S, Gibson J, Généreux M (2018). Public health emergency preparedness: a framework to promote resilience. BMC Public Health 5 déc.

[49] Smith R, Allin S, Rosella L, Luu K, Thomas M. Profiles of Public Health Systems in Canada: Ontario. Volume 42. National Collaborating Centre for Healthy Public Policy; 2021.

[50] Arpin E, Smith R, Cheung A, Thomas M, Luu K, Li J et al. Profiles of Public Health Systems in Canada: Québec [Internet]. National Collaborating Centre for Healthy Public Policy; 2022. Disponible sur: https://ccnpps-ncchpp.ca/docs/2022-Profiles-of-Public-Health-Systems-in-Canada-Quebec.pdf

[51] Smith RW, Allin S, Luu K, Cheung A, Thomas M, Li J, National Collaborating Centre for Healthy Public Policy. Profiles of Public Health Systems in Canada: Nova Scotia [Internet]. Montreal, Quebec: ; 2022 p. 34. Disponible sur: https://ccnpps-ncchpp.ca/docs/2021-Profiles-of-Public-Health-Systems-in-Canada-Nova-Scotia.pdf

[52] Smith R, Allin S, Luu K, Jarvis T, Thomas M, Li J, National Collaborating Centre for Healthy Public Policy. Profiles of Public Health Systems in Canada: Alberta [Internet]. ; 2022 p. 44. Disponible sur: https://ccnpps-ncchpp.ca/docs/2022-Profiles-of-Public-Health-Systems-in-Canada-Alberta.pdf

[53] Marchildon GP (2015). The crisis of regionalization. Healthc Manage Forum.

[54] Rechel B. How to enhance the integration of primary care and public health? Approaches, facilitating factors and policy options. London; 2020 p. 26. (European Observatory on Health Systems and Policies). Report No: 34. Disponible sur: https://apps.who.int/iris/handle/10665/330491 32073809

[55] Breton M, Denis JL, Lamothe L (2010). Incorporating public health more closely into local governance of health care delivery: lessons from the Québec experience. Can J Public Health.

[56] Agence de santé publique du Canada (2008). Division du perfectionnement de la main-d’oeuvre. Compétences essentielles en santé publique au Canada: version 1.0.

[57] DiRuggiero E, Bathia D, Umar I, Arpin E, Champagne C, Clavier C et al. Governing for the Public’s Health: Governance Options for a Strengthened and Renewed Public Health System in Canada [Internet]. National Collaborating Centres for Public Health; 2022 p. 104. Disponible sur: https://nccph.ca/images/uploads/general/OCPHO-Report-Governance-2022-En.pdf

[58] Carlson V, Chilton MJ, Corso LC, Beitsch LM (2015). Defining the functions of public health governance. Am J Public Health avr.

