# Supplementary material for: Prioritization of public health financing, organization, and workforce transformation: a Delphi study in Canada
Source: BMC Public Health. 2023 Mar 22;23:544. doi: 10.1186/s12889-023-15373-9 (PMC10031161; doi:10.1186/s12889-023-15373-9)
Supplement: Supplementary file 1 — Supplementary Material 1 [file 12889_2023_15373_MOESM1_ESM.docx]

**Supplementary Material**

**Prioritization of Public Health Financing, Organization, and Workforce Transformation: A Delphi Study in Canada**

**Supplementary Material 1: Delphi Questionnaire**

**Round 1:**

Thank you for agreeing to participate in this Delphi survey on your perceptions of the priorities for changes to public health systems.

While several studies are underway to gain insight into the impacts of recent reforms on provincial/territorial public health systems, and the public health responses to the COVID-19 pandemic, we precisely aim to understand the views of public health leaders on the key issues surrounding public health financing, organization, interventions, and workforce. The uniqueness of this survey is analogous to a prioritization exercise where we intend to generate knowledge relevant for public health communities during and in the recovery of the COVID-19 pandemic. The results of this prioritization exercise will help us to design a discrete choice experiment to understand how public health leaders make trade-offs between these key issues.

Ultimately, we aim for the findings from this research to help inform the (re)design of public health systems in the context of the COVID-19 pandemic.

Therefore, we are seeking your view on the importance of characteristics of your public health system. While most of these characteristics will be relevant to the provincial or territorial level, some may also relate to your regional and local level, and we ask you to please keep this multidimensional level in mind.

Below is a list of propositions that you will rank from not important (1) to very important (5).

**Table S1**: Rating exercise in the Delphi

|  | Perceptions on the importance of changes to your provincial or territorial public health system | Not important |  |  |  | Extremely important |
| --- | --- | --- | --- | --- | --- | --- |
|  | Questions on financing |  |  |  |  |  |
| 1 | Public health budget in your province or territory (e.g., increase, decrease or stability) | 1 | 2 | 3 | 4 | 5 |
| 2 | Source of public health financing (e.g., mostly federal or mostly provincial or mostly municipal) | 1 | 2 | 3 | 4 | 5 |
| 3 | Time frame for spending (e.g., restricted to a fiscal year or possible to use beyond a fiscal year) | 1 | 2 | 3 | 4 | 5 |
|  | Questions on organization |  |  |  |  |  |
| 4 | Centralization or decentralization (e.g., less or more public health structures in your province or territory) | 1 | 2 | 3 | 4 | 5 |
| 5 | Integration or separation of public health with other health sectors (e.g., with primary care) | 1 | 2 | 3 | 4 | 5 |
| 6 | Higher or lower number of institutions specializing in particular public health functions (e.g., some institutions focus on surveillance, other on promotion) | 1 | 2 | 3 | 4 | 5 |
|  | Questions on workforce and interventions |  |  |  |  |  |
| 7 | Disciplinary skill mix of public health human resources (e.g., concentration of some discipline or more multidisciplinarity) | 1 | 2 | 3 | 4 | 5 |
| 8 | Health protection interventions directly related to the COVID-19 pandemic (e.g., surveillance, case and contact management, infection prevention and control, risk communication) | 1 | 2 | 3 | 4 | 5 |
| 9 | Public health interventions beyond the health protection interventions directly related to the COVID-19 pandemic (e.g., prevention of other infectious and non-communicable diseases, health promotion, environmental health protection and promotion) | 1 | 2 | 3 | 4 | 5 |

Legend: 1 to 5 scale range from “not at all important”, “slightly important”, “moderately important”, “very important” to “extremely important”.

**[next page on Qualtrics website]**

We developed the list you rated after a comprehensive literature review and in consultation with public health practitioners and knowledge users. We however want to ensure that we capture all the potential priorities in the public health community before moving to the second round of the Delphi.

What are other characteristics related to changes in public health financing, organization, interventions, and workforce not itemized previously that should be added to our list for future consideration? (up to 3)

1.

2.

3.

Please provide a brief rationale for your choices.

**[next page on Qualtrics website]**

Thank you again for your time. You will receive an invitation to participate in the second round of this Delphi survey on Day Month Year.

**Rounds 2 to 3:**

In the next rounds, an individualized survey will be sent to the respondents. The survey will summarize all respondents’ ratings as well as remind the respondent their ratings during the previous round. Then the table of propositions will be displayed again, and the respondent will have an opportunity to reassess their rating based on the previous round results.

**[In the third round only]**

Thank you again for your time. We will be sure to communicate our findings widely, in aggregate only, with the help of our knowledge users’ network. You will also receive the report on the findings by email.
